# Effect of Diet on CPFAs Used as Markers in Milk for the Detection of Silage in the Ration of Dairy Cows

**DOI:** 10.3390/foods14030476

**Published:** 2025-02-02

**Authors:** Federico Fava, Demian Martini-Lösch, Giovanni Peratoner, Peter Robatscher, Aldo Matteazzi, Evelyn Soini, Andreas Österreicher, Simon Volgger, Rafael de Andrade Moral, Matteo Mario Scampicchio, Daniela Eisenstecken, Elena Venir

**Affiliations:** 1 Laimburg Research Centre, Laimburg 6—Pfatten/Vadena, 39040 Auer/Ora, BZ, Italy; federico.fava@laimburg.it (F.F.); demian.martini-loesch@laimburg.it (D.M.-L.); peter.robatscher@laimburg.it (P.R.); aldo.matteazzi@laimburg.it (A.M.); evelyn.soini@laimburg.it (E.S.); daniela.eisenstecken@laimburg.it (D.E.); elena.venir@laimburg.it (E.V.); 2 Sennereiverband Südtirol, Via Galvani 38, 39100 Bozen/Bolzano, BZ, Italy; andreas.oesterreicher@sennereiverband.it; 3 BRING Beratungsring Berglandwirtschaft, Via Galvani 38, 39100 Bozen/Bolzano, BZ, Italy; simon@milchviehberatung.it; 4 Department of Mathematics & Statistics, Maynooth University, W23 F2H6 Maynooth, Ireland; rafael.deandrademoral@mu.ie; 5 Faculty of Agricultural, Environmental and Food Sciences, Free University of Bozen-Bolzano, Piazza Università 5, 39100 Bozen/Bolzano, BZ, Italy; matteo.scampicchio@unibz.it

**Keywords:** hay milk, grass silage, maize silage, dihydrosterculic acid, lactobacillic acid, food authentication

## Abstract

In hay milk production, fermented feed, like silage, is forbidden. This study aims to reveal the presence of silages made from maize or grass in the diet of dairy cows through the detection of cyclopropane fatty acids (CPFAs) in their milk. It also investigates how CPFAs in their milk declines when the diets of the cows are transitioned from one containing silage to one that does not include silage. CPFAs were quantified in silages collected on the farm, and the relationship between the dietary intake of CPFAs from silages and the marker concentration in milk was investigated. Except for one sample (below LOQ), CPFAs were never detected in hay milk, while they were found in 98% and 85% of milk samples obtained from cows whose diet included maize or grass silage as the only fermented component, respectively. CPFAs were found to still be detectable in milk 56 days after the removal of maize silage from the diet, while they were no longer detectable about three weeks after removing grass silage from the ration. A quantitative positive relationship was detected between CPFAs content in the milk and the dietary intake of CPFAs from silages. CPFAs can be regarded as reliable markers to detect the occurrence of silages in the ration, but it is more effective for maize than for grass silage.

## 1. Introduction

### 1.1. Hay Milk Definition and Relevance in the Agricultural Practice

Markers in foods are useful tools to provide proof of respecting the production specifications (both mandatory or internal guidelines), and often support the authentication process of many foods, including traditionally protected products. Among these products, hay milk, over the last eight years, has gained consumers’ appreciation and has found commercial success in Europe. This milk type requires a restricted diet for cows, as silage and any other kinds of fermented fodders are forbidden, and roughage must make up at least 75% of the dry yearly ration [1]. In 2017, hay milk accounted for 3% of the European milk market, 15% of the Austrian milk market, and up to 40% in some regions in the Alps such as Vorarlberg and Tyrol (Austria) [2]. In South Tyrol, the study area of this paper, being the northernmost region of Italy dominated by an alpine landscape, dairy farming is characterised by a small-scale agricultural structure and mountain grassland [3]; here, hay milk accounted for 26.4% of the total milk production in 2023 [4].

### 1.2. Hay Milk Authentication and the Role of CPFAs

Several studies have been conducted to differentiate milk types based on the feeding regimen [5,6,7]. The most important analytical approach used to assess cows’ diets is the analysis of milk lipidome, being a pool of relevant molecules. Recently, two classes of markers, namely cyclopropyl and ω-cyclohexyl fatty acids, have been identified [8] as potential tools for milk and cheese authentication. Further investigations confirmed the strict correlation between the inclusion of maize silages in the feed ration and the presence of cyclopropyl fatty acids (CPFAs) in milk fat [9,10]. Indeed, CPFAs have been studied in dairy products [11] and used as target markers since 2016 for the differentiation between Parmigiano Reggiano and Grana Padano cheeses. Production regulations for Parmigiano Reggiano do not allow the use of any silage, while for Grana Padano, the use of maize silage in the diet of the cows is permitted.

Silages naturally contain lactic acid bacteria (LAB) which accumulate CPFAs in their phospholipid membranes under stress conditions [10]. These molecules include two main fatty acids: dihydrosterculic acid (DHSA), which is reported to be the most abundant in LAB membranes [11], and lactobacillic acid (LBA). These fatty acids are produced as a post-synthetic modification of the phospholipidic membrane of LAB [12,13,14], which are abundant during fermentation processes. This modification is caused by different environmental stresses, such as a change in pH, in osmotic pressure, or in temperature [15,16,17]. In ensiled substrates, pH was found to be highly inversely correlated to CPFAs, indicating that pH is an especially key factor in stressing LAB; moreover, the anaerobic environment during the ensiling process would lead in turn to increased LAB stress, and therefore CPFAs synthesis [15]. The synthesis of the cyclopropane ring improves membrane resistance and fluidity, and is one of the most important adaptive microbial responses to stress exposure [13,18,19]. CPFAs content in dairy products is solely due to the use of silage feeding and not to bacterial fermentation in food, e.g., that in yoghurt or cheeses [11]. As reported by the authors [9], Grana Padano cheese always tested positive for the presence of CPFAs, with a variable amount ranging from 300 to 830 mg kg^−1^ of fat and a mean content of 540 ± 110 mg kg^−1^ of fat among the samples analysed. Interestingly, the authors did not detect CPFAs in sheep, yak, and goat milk and sheep and goat cheeses [11], but CPFAs have been detected in most ovine cheese samples, both from hay and silage-based diets [20]. Moreover, CPFA levels in cheese fat from hay feeding were found to be positively correlated to the total trans-monounsaturated fatty acids (MUFAs) and n-6 polyunsaturated fatty acids (PUFAs), whereas they were negatively correlated to cis-MUFAs, odd- and branched-chain fatty acids (i.e., C13:0 anteiso, C16:0 iso, and C17:1), and C22:5n-3, which are mainly associated with a low starch intake [20]. Lolli et al. [21] reported that the cyclopropane ring in fatty acids is resistant to gastrointestinal digestion since it is not degraded during in vitro digestion and, through in vitro simulation, it has been demonstrated that the rumen conditions should not affect the CPFAs content, so that only the ensiling environment is responsible for CPFAs synthesis and accumulation [15].

It should be considered that CPFAs are also present in some plants [22], especially in tropical plant seeds, and they are particularly abundant in *Sterculia foetida* seed oil, from which the name sterculic acid comes from [23]. However, the presence of CPFAs in any of the endemic plants commonly found in the meadows and pastures of South Tyrol is not reported. The only family (Malvaceae) of which species occur in South Tyrol, and which are suspected to possess CPFAs, are not part of the usual botanical composition of grassland managed for forage production, as reported by several publications describing the botanical composition of the respective grassland plant communities and vegetation types [24,25,26], or listing the plant communities in which species of the Malvaceae family occur [27].

In the literature, CPFAs concentration in dairy products ranges between 200 and 1000 mg kg^−1^ fat [21], and from 60 to 800 mg kg^−1^ fat in meat and fish [28,29], indicating that the dietary intake for humans may not be negligible [30,31,32]. Indeed, the plasmatic concentration in humans of CPFAs, mainly DHSA, is reported to be affected by the consumption of CPFAs-rich foods [33].

### 1.3. Knowledge Gaps Concerning the Use of CPFAs as Authentication Tools

The existing research has primarily focused on detecting the occurrence of maize silage in the cows’ ration through the analysis of CPFAs in milk and cheeses [9,11].

To the best of our knowledge, only two studies have explored CPFAs concentration in grass silage [15,34], showing lower levels of CPFAs in grass silages than in maize silages. However, in mountain regions like South Tyrol, grass silage produced on farm is often part of the diet of dairy cows, and no studies, so far, have investigated the link between the occurrence of CPFAs and farm management practices, specifically related to the quantitative occurrence of silages in the feed ration. In our previous research [35], we demonstrated that CPFA levels could differentiate milk from cows fed maize silage, grass silage, and no silage, although this was based on a limited dataset (n = 27), being part of the sample pool (n = 242) used for the present study. A further study, including the whole pool of samples and using quantitative ^1^H NMR spectroscopy [36], detected CPFAs in 97% of maize silage-fed and 77% of grass silage-fed milk samples, while no CPFAs were detected in hay milk samples.

Moreover, the current EU regulation for hay milk [1], requires a two-week period from the suspension of feeding any fermented feedstuff prior to labelling milk as hay milk or hay milk-derived products. Nevertheless, to date, we are not aware of analytical evidence supporting the adequacy of this two-week period. Therefore, experimental validation of this regulatory requirement is needed to ensure the reliability of hay milk labelling and production practices.

### 1.4. Aim of the Study

The aim of this study was to (i) describe the occurrence of CPFAs in both maize and grass silage produced on-farm, (ii) assess the reliability of detecting both grass and maize silage feeding in dairy cows through the presence of CPFAs in milk, (iii) quantify the time required for CPFAs to disappear from milk following the suspension of silage feeding, depending on the type of silage fed, and (iv) investigate the quantitative relationship between the dietary intake of CPFAs from silages and their concentration in milk, to assess the reliability of hay milk authentication on an analytical basis.

## 2. Materials and Methods

### 2.1. On-Farm Sampling Plan

A call for interest was made to voluntarily recruit farms into taking part in the study. A representative number of 42 candidate dairy farms in South Tyrol (NE Italy) were selected and interviewed about their operational structure and the composition of their feed ration. This was conducted by Sennereiverband (the local dairy association) and BRING (the local extension service for mountain agriculture) among their member farms in South Tyrol. The sampling plan was designed to encompass, as far as possible, the commonly occurring silage types and proportions in the diet in the study area. Farms with either grass silage or maize silage as the only fermented feedstuff in their feed ration, along with four hay milk farms, were involved in the study. For all selected farms, the diet fed to milk cows at lactation peak was assessed. To this aim, the amount of each ration component in the diet was recorded on-farm, and the dry matter proportion of silage was assessed using dry matter reference values associated with each ration component. The estimate of the amount of concentrate was adjusted based on the mean lactation stage of the herd. This assessment of the ration was repeated for each farm and sampling phase in order to account for diet changes over time. It was ensured that the farms had their own milk tank and that no other fermented feed type (e.g., brewer’s spent grains, often included in the diet in this region) was fed, to exclude any possible additional source of the marker.

### 2.2. Experimental Design

Two different experiments were conducted.

In the first experiment, the trend in the content of CPFAs in milk over time was investigated after ceasing to include silages (grass or maize) in the diet in order to quantify the time taken for CPFAs to disappear in the milk after silage removal from the ration. To this aim, six farms (three for maize silage and three for grass silage) were sampled. Each farm switched from a ration with silage to a diet without silage. At each farm, five samples were taken by means of sequential sampling, with an interval of two weeks between sampling events.

In the second experiment, CPFAs were quantified in milk produced following three different diets for lactating cows, with the aim to investigate the relationship between the dietary intake of CPFAs with the silages and the occurrence of CPFAs in the milk. Three milk types were investigated: hay milk (HM), milk produced with a ration including grass silage as the only fermented feedstuff in the diet (GSM), and milk produced with a ration including maize silage as the only fermented feedstuff in the diet (MSM). Sampling was repeated for two years (winter 2019 to summer 2021) and during two different sampling phases within each year, representing the summer ration (May to September) and the winter ration (November to March). For each sampling phase within each year, eight farms for GSM and MSM were selected to cover the range of silage proportion in the diet of dairy cows in the study region, resulting in a range of 15 to 53% for grass silage, and of 7 to 40% for maize silage on a dry matter basis (Figure S1). The choice of the farms to be included in the sampling plan was led by the aim to obtain a good coverage of the whole gradient, stretching from the lowest to the highest value found for each silage type among the available farms. Here, an exception was made to the rule of feeding exclusively maize or grass silage as the only fermented feedstuff in the diet for one farm being sampled in the winter seasons 2019–2020 and 2020–2021, which fed mainly maize silage (3.5 maize silage/1 grass silage). As these observations were relevant to improve the gradient continuity of silage proportion in the diet, this departure from the general rule was allowed based on the knowledge that maize silages have, based on median values, a CPFAs content about sevenfold that of grass silages (225.9 vs. 33.2, mg kg^−1^ of fat) [34]. Therefore, it was assumed that, in this case, maize silage was the silage type overwhelmingly contributing to the overall dietary CPFAs intake.

The sampling plan resulted in quite an even distribution of the participating farms within the region (Figure S2) and a broad range of farm characteristics. They were located at elevations ranging between 540 and 1470 m a.s.l., had a median number of 25 cows per farm (range: 13–110) and milk yields ranging between 6000 and 10,000 kg year^−1^ (median: 8000 kg year^−1^) (Table S1). As a control, four hay milk farms that fed cows according to the EU regulation [1] for hay milk production were selected. Within each of the sampling phases, bulk milk was sequentially sampled weekly, over a period of three weeks. In summer 2020, one farm including maize silage in the diet was sampled only twice. The samples were collected in the morning directly from the milk tank, i.e., as a mixture of evening and morning milk. The milk was stirred well and drawn from the tank with a liquid sampler. The samples were transported within two hours after sampling to the lab in a cool box at 4 °C and then stored at −80 °C in the freezer. A total of 244 milk samples were collected for this experiment. Additionally, on the first sampling event of each sampling phase, 500 g of the mixed ration, as well as 400 g of the silage being included in the ration, were sampled. For farms equipped with a forage mixer, the mixed ration was directly sampled on site. If a forage mixer was not available, the individual components of the ration were collected separately and subsequently mixed according to the ration calculations. The silage samples were taken by hand at the current break-off point or opening in the silo so that the sample corresponded exactly to what the farmer was currently feeding. To obtain a representative mixture, samples were taken from five different randomly chosen positions of the accessible silage surface and then merged into one sample. All feed samples were frozen and stored at −40 °C until analysis. The silage samples were analysed to assess both their quality and the CPFAs content. A total of 67 silage samples, of which 35 were grass and 32 maize silage samples, were collected, analysed, and used for data analysis.

### 2.3. Chemicals and Reagents

Methanol was purchased from VWR (Radnor, PA, USA), and n-pentane was obtained from Fluka Analytical (Honeywell International Inc., Charlotte, NC, USA). n-Heptane was purchased from Sigma Aldrich (St. Louise, MO, USA), and potassium hydroxide was purchased from Carlo Erba Reagents srl (Milano, Italy, IT). Sodium sulphate was purchased from Titolchimica (Rovigo, Italy, IT) and the CPFA Methyl cis-9,10-Methyleneoctadecanoate (dihydrosterculic acid DHSA methyl ester, purity > 98%) was obtained from Larodan AB (Solna, Sweden, SWE).

### 2.4. Sample Preparation and Analysis via GC-MS

#### 2.4.1. Milk Fat Extraction

The milk samples were thawed in a water bath at 40 °C for 2 h. The fat was separated following a modified method based on Feng, as described before [35,37]. A volume of 20 mL of milk was added in a 50 mL conical plastic tube and centrifuged at 12,000 rpm (17,800× *g*) for 30 min at 4 °C. After centrifugation, the fat cake (top layer) was transferred into a 15 mL conical plastic tube and stored over night at −80 °C. The fat was resuspended in a volume of 10 mL of a 9:1 (*v*/*v*) n-pentane/methanol, then vortexed for 2 min at room temperature and kept in a room temperature ultrasound bath (45 kHz) for 5 min, shaken for 5 min with a MultiRotator (PTR-60 Grant Instruments, Royston, UK), then vortexed again for 2 min with a final centrifugation at 4000 rpm (1900× *g*) for 2 min at room temperature. The organic phase was transferred into a dark glass vial and flushed with N_2_ until dryness. The fat was stored at −80 °C until transesterification.

#### 2.4.2. Silage Fat Extraction

The silage samples were freeze-dried, ground in an analytical mill (IKA A11 basic—IKA^®^, Staufen, Germany, DE) and extracted with n-pentane using the semi-automatic extractor (Velp SER 148, Velp Scientifica SRL, Usmate Velate, Italy).

#### 2.4.3. Milk Fat Transesterification

Transesterification was carried out according to ISO 15884:2002 IDF 182 [38]. Milk fat (100 mg ± 5 mg) was dissolved in 5 mL of heptane. Then, 0.2 mL of a KOH 2M in methanol was added, and the solution was agitated to ensure thorough mixing and left resting for few minutes. Then, 0.5 g of sodium sulphate were added following a brief agitation (30 s). The samples were centrifuged at 2000× *g* for 5 min at room temperature, and the supernatant, diluted 1:10 with heptane, was used for analyses.

#### 2.4.4. Silage Fat Transesterification

Silage fat was transesterified with a modification of the ISO 15884:2002 IDF 182 [38] method in order to avoid the hydrolysis of the CPFAs ring [14]. A mass of 50 mg ± 5 mg was dissolved in 5 mL heptane. Then, 0.2 mL of KOH 2M in methanol was added. The solution was agitated for few minutes and quenched by adding 0.5 g sodium sulphate. After a brief agitation, it was centrifuged at 2000× *g* for 5 min at room temperature. The supernatant, diluted 1:10 with heptane, was used for analyses. It should be noted that acid-catalysed methylation usually adopted to generate methyl esters, which also convert free fatty acids possibly present in silages, might cause the opening of cyclopropane ring during the conversion to FAME [14], thus leading to an underestimation of CPFAs. For this reason, we opted for a base-catalysed method for the methyl esterification of phospholipids and triglycerides using potassium methoxide in anhydrous methanol.

#### 2.4.5. Analysis of Cyclopropane Fatty Acids

The GC-MS analysis was carried out on a Shimadzu GC MS-QP2010 SE (Kyoto, Japan) equipped with an autosampler, a split/splitless injection port, a GC oven, and a single quadrupole mass spectrometer. Each sample of the repeated measure was analysed in a single run. A total of 1 µL was injected using a split ratio of 1:10. For the calibration curve in the matrix, hay milk fat was used for milk, and grass fat before the ensiling process was used for silage. Helium was used as a carrier gas with a flow rate of 1 mL min^−1^, and a low-polarity SLB-5 ms column (30 m × 0.25 mm i.d. × 0.25 µm) (Supelco, Bellefonte, PA, USA) was used for the chromatographic separation of the analyte. The run was conducted following a modified temperature programme, as described before in [35]. The temperature was kept at 40 °C for 5 min, increased to 280 °C at a rate of 10 °C min^−1^, and held for 10 min. The injector temperature and transfer line temperature were maintained at 280 °C, and the ion source temperature at 230 °C. The mass spectra were acquired in full scan mode (mass range 40–500 *m*/*z*) and in SIM mode (using 55 *m*/*z* as quantifier, 69 and 278 *m*/*z* as qualifiers). The quantification of CPFAs in the samples was assessed by comparing the peak area of the samples with the peak area of known amounts of the CPFAs standard, considering the matrix effect by haymilk spiked with DHSA following extraction and transesterification. For milk fat analysis, the limit of detection (LOD) of the method was 8 mg DHSA kg^−1^ of fat and the limit of quantification (LOQ) was 26.0 mg DHSA kg^−1^ of fat. For silage fat analysis, the LOD was 7 mg kg^−1^ fat, and the LOQ was 25 mg kg^−1^ of fat. For statistical analysis, CPFAs values between the LOD and LOQ were used and assigned to each sample by the software, whilst values below the LOD were manually assigned by the visual inspection and integration of the peak in the chromogram. The linear range was from 25.0 µg L^−1^ to 1500 µg L^−1^. Recovery of DHSA in spiked fat was 101.5% (0.2 RSD%). Intraday repeatability was of 3.3, 5.4, and 2.5 RSD% for 80, 400, and 1000 µg DHSA L^−1^, respectively.

### 2.5. Feed Analysis

The dry matter content was determined by oven-drying the forage samples at 60 °C until weight constancy. Weende analysis [39] was performed to determine the physico-chemical properties of silage samples. The neutral detergent fibre (NDF) and acid detergent fibre (ADF) of the mixed feed rations were determined through Van Soest analysis [39]. For the NDF analysis, both alpha-amylase and sodium sulphite were used. The analysis of mineral elements was performed according to the VDLUFA Method Book III [39] (Method 11.1.2:1983), using inductively coupled plasma–optical emission spectroscopy (Agilent, Santa Clara, CA, USA).

### 2.6. Computation of Metrics to Quantify the CPFAs Intake with Silage in the Diet

The dietary CPFAs taken up by the lactating cows were indirectly quantified by means of proxies (1 and 2) or directly quantified in five different ways. These were, in order of increasing complexity, availability of information and analytic effort:

1. The percentage of silage in the diet on a dry matter basis (Figure S4a), assessed as described above.
2. The mean daily silage intake per cow on a dry matter basis (Figure S4b), assessed in the same way.
3. The daily dietary CPFAs intake from the silage per cow (Figure S4e). To this aim, the CPFAs concentration in the silage’s fat (determined as described above) was multiplied by the mean daily silage intake per cow and by a reference value of lipid content of silages. This was estimated using reference values [40]: for maize silage, an average of the values between the beginning and end of waxy maturity (2.65%, range: 2.4–2.8%) was used. For grass silage, the average of values given for all phenological stages (3.02%, range: 2.9–3.1%) was used. For mixed silages, a weighted average based on the percentage of silage as declared by the farmers was calculated.
4. The dietary CPFAs intake from the silage per kg of milk produced (Figure S4f). The daily CPFAs intake per cow was divided by the mean daily milk yield. The latter was obtained by dividing the mean annual milk yield of the respective farm (provided by the milk plant and rounded at 500 litre, Figure S4b) by a theoretical lactation period of 305 days.
5. The dietary CPFAs intake from the silage per kg of fat produced with the milk (Figure S4g). To this aim, the daily CPFAs intake per cow was divided by the product of the mean daily milk yield and the milk fat content at the time of the milk sampling event (Figure S4d).

### 2.7. Statistical Analysis

The effect of the time since suspension of silage feeding and of the silage type on the CPFAs content in the milk was analysed by means of linear mixed models, taking into account the main terms and their interaction, as well the repeated measurements over time at the same farm with a first-order autoregressive covariance structure. The time since suspension of silage feeding was modelled by polynomial regression. The statistical model was built stepwise forward, starting from a baseline model accounting only for a first-degree polynomial of the time since suspension of silage feeding, and adjusting the polynomial degree first for the main term and then for the interaction with the silage type using a type I sum of squares, Akaike’s information criterion as an indicator of model fit, and maximum likelihood as the estimation method. The terms were stated in the order given above. For the final model, restricted maximum likelihood was used as the estimation method.

The effect of the seasonality and the silage type on the CPFAs content in silage was investigated by linear models accounting for the sampling season, the silage type, and their interaction, as well as for the sampling year, which was considered a random effect. The dependent variable was square root-transformed to fulfil the assumptions for the analysis (normal distribution of residuals, homoscedasticity). All analyses described so far were checked by means of diagnostic plots [41].

The correlation between the variables used to indirectly or directly quantify the dietary CPFAs intake from silages was explored by nonparametric Spearman’s rank correlation, as an apparent nonlinear relationship between the variables was detected during a graphical data exploration. Estimation of the 95% confidence interval of the correlation coefficients was computed based on Fisher’s r-to-z transformation. The estimation of the standard error was computed based on the formula proposed by Fieller, Hartley, and Pearson.

Due to violations of the normal distribution of the residuals, the quantitative effect of the dietary CPFAs intake from silages on the CPFAs content in the milk was analysed by means of generalised additive models for location scale and shape [42], using a zero-adjusted inverse Gaussian distribution with a log link function for the mean (µ) and dispersion (σ) parameters and a logit link function for the zero-inflation (ν) parameter. For this analysis, the observations at farms not feeding any kind of silages (hay milk farms) were randomly assigned to the observations pool of one of the two silage types. Three observations concerning a farm feeding maize silage showing anomalous CPFAs content in the silage (1292.3 mg kg^−1^ fat) were not used for the analysis. This value represented, indeed, an increase of 76% in comparison to the preceding value (735.7 mg kg^−1^ fat), representing a weak data basis to extend the prediction to this region of the CPFAs content in the silage (see the three highest values on the x-axis in Figure S5). As accounting for the repeated measurements over time at the same farm within a sampling period by means of a compound symmetry covariance structure led to a lack of convergence, the analysis was conducted using means over the repeated measurements events. The analysis started from a full model, including the µ part of the model, the year, the season, the silage type, the dietary CPFAs intake from silages, the interactions between the latter and silage type, and the interaction between the season and silage type. The σ part of the model accounted for the sampling period, whilst the ν part of the model accounted for the dietary CPFAs intake from silages. The model was further stepwise backward developed by dropping the model terms not leading to an improvement of the model, as ascertained by fitting all possible models by dropping the terms one at a time and computing the changes in fit. Model goodness-of-fit was assessed by means of worm plots [43].

For all statistical analyses, an effect associated with a *p*-value < 0.05 was considered statistically significant.

Moreover, an exploratory PCA was conducted on means of the values obtained by repeated measurements over time at the same farm within the same observation period, taking into account the daily intake of selected feed components (crude ashes, crude protein, neutral detergent fibre, acid detergent fibre, P, and Fe), computed as a product of their content in the total ration fed to the cows at the time of milk sampling and of the mean daily forage intake, the milk yield, the prevailing breed kept at the farm, the purchase of off-farm silages, the use of silage starters, the occurrence of grazing at the time of milk sampling, the milk fat content, the CPFAs content in the silages included in the ration, the daily silage intake, the dry matter percentage of silage in the ration, the daily ration intake, the mean daily CPFAs intake with the silages in the ration, the mean CPFAs intake per kg of milk, the mean CPFAs intake per kg of fat produced with the milk, and the CPFAs concentration in the milk. Prior to the analysis, the data were z-standardised to achieve a distribution with mean = 0 and standard deviation = 1 for each parameter. A singular value decomposition algorithm was used on variance–covariance matrices.

The descriptive statistics and the correlation analysis were performed by IBM SPSS Statistics (Version 29.0.1.0), and all other analyses with were performed with R (RStudio 2023.09.0+463). In R, the *lme* function [44] within the package *nlme* was used for the linear mixed models analysis, the package *effects* was used [45] to plot the function obtained with *lme*, the package *gamlss* [46] was used for the analyses by means of generalised additive mixed model for location, scale and shape, its function *wp* was used for the evaluation of the adequacy of the distribution chosen for the analysis via worm plots, the function *drop1* was used to compute likelihood ratio tests between models when dropping one predictor at a time from each of the parameters in the model, and the package *ggplot2* [47] was used to plot the results of the analysis made with *gamlss*. The PCA was performed with the software PAST (Version 4.09) [48] Object labels, where necessary, were edited with a free, open source vector graphics editor (Inkscape, version 1.3).

## 3. Results and Discussion

### 3.1. Changes in CPFAs Concentration in Milk After Silage Suspension

The CPFAs concentration in the milk was found to decrease over time, following a suspension of silage feeding in the diet of dairy cows, according to a third-degree polynomial, with two different courses for maize and grass silage (Table 1).

**Figure 1:**
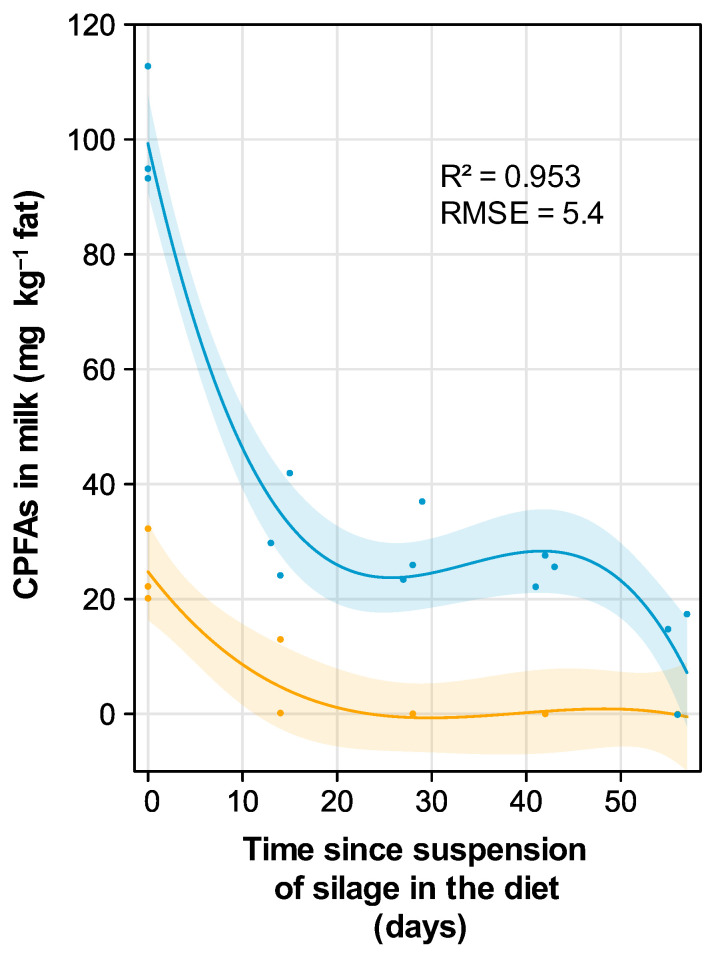
Change in CPFAs concentration in the milk over time following the suspension of maize and grass silages in the diet of dairy cows. Predicted mean values (solid lines) and 95% confidence intervals for the true mean (shaded ribbons) are shown against the observed values. Orange = grass silage, blue = maize silage. R^2^ = coefficient of determination, RMSE = root mean square error.

For milk obtained from cows fed with grass silage, CPFAs were initially detected at a relatively low concentration (around 20 mg kg^−1^ fat) at the time of suspension of silage in the diet. The CPFAs content rapidly decreased and became virtually undetectable approximately three weeks after suspension (Figure 1). In contrast, in milk from cows fed with maize silage, the CPFAs concentration at the time of silage suspension was about fivefold higher than that observed for the diet containing grass silage. Following a very quick decrease over the first 20 days, the values remained at low levels for a further 25 days. Eight weeks after silage suspension, the CPFAs concentration was close to zero. For both silage types, the time needed for the marker to become undetectable exceeded the two-week period following the suspension of silage feeding specified in the hay milk product regulations. This discrepancy implies, on one hand, that using CPFAs as a marker could lead to false positives in verifying compliance with hay milk standards, especially for milk produced by cows fed with maize silage. On the other hand, this makes this marker very conservative for preliminary screening purposes.

Concerning the question about the time needed to detect CPFAs in the milk after the introduction of maize silage in the cow’s diet, we also report here the results of an explorative investigation performed weekly at one farm in one season and one year only, and thus without replicates. CPFAs were detected from the third day after silage introduction in the diet and reached their maximum concentration within 10 days (Figure S3), exhibiting moderate fluctuation over the 54 days of sampling time. This leads to the hypothesis that the appearance of CPFAs in the milk is a very rapid process. However, due to the lack of replicates, this needs further investigation.

### 3.2. Variability of CPFAs in Silages and Milk

**Figure 2:**
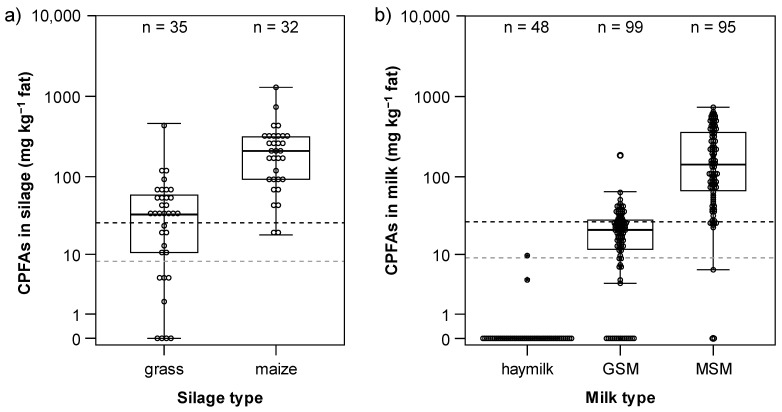
Distribution of CPFAs content (**a**) in the silage depending on the silage type and (**b**) in the milk depending on the milk type (right). Hay milk = production according to the EU regulation 2016/304; GSM = diet including grass silage; MSM = diet including maize silage. The grey dashed line represents the limit of detection (LOD) for silage fat, 7 mg CPFAs kg^−1^ and for milk fat, 8 mg kg^−1^ CPFAs, the black dashed line represents the limit of quantification (LOQ) for silage fat, 25 mg CPFAs kg^−1^ and for milk fat, 26 mg kg^−1^ CPFAs. One was added to each value to allow for visualisation in a logarithmic scale.

In the second experiment, the CPFAs content in silages was found to be strongly affected by the silage type (F = 40.42, *p* < 0.001), whilst no effect was detected for the seasonality (F = 0.022, *p* = 0.883) or the interaction between silage type and seasonality (F = 0.162, *p* = 0.689). However, a large variability in CPFAs content was observed for both the grass and the maize silages collected on farm (Figure 2a, Table 2). The CPFAs concentration ranged widely from 0.0 to 460.8 mg kg^−1^ of fat for grass silage (mean: 49.3 mg kg^−1^ of fat), and was considerably higher for maize silage, the CPFAs content of which ranged between 18.1 and 1292.3 mg kg^−1^ of fat (mean: 250.5 mg kg^−1^ of fat). This variability was shown in laboratory-produced silages to be partly affected (depending on the ensiled sample) by the incubation temperature, whilst both laboratory-produced and on-farm silages exhibited a correlation of CPFAs with several forage quality parameters, of which the concentration of acetic acid was the most consistent [34].

The high variability in the CPFAs will have a considerable impact on the determination of effective dietary intake of CPFAs when estimating it through simple silage intake; even if the diet contains high proportions of grass silages, the actual dietary intake of CPFAs may still be absent. In the case of maize silages, the very wide range of CPFAs content (18–1293 mg kg^−1^ of fat) can be expected to cause considerable errors when attempting to use the daily silage intake as a proxy for the dietary intake of CPFAs.

For grass silage, 77.1% of the samples were positive for the occurrence of CPFAs: the percentage of samples exhibiting CPFAs content exceeding the LOQ was 60.0%. A total of 11.4% of the samples contained no CPFAs, a further 11.4% had contents lower than the LOD, and 17.1% of the samples lied between the LOD and the LOQ. For eight different grass silages (ryegrass, alfalfa, wheat, and a mix of cereals), Lolli et al. [15] reported CPFAs contents of between 2.6 and 123.4 mg kg^−1^ dry matter in samples positive for the occurrence of CPFAs, with the alfalfa silage showing the lowest value and the mix of cereal species the highest one. Four of the investigated samples (33.3%) exhibited CPFAs values lower than the LOD, which is roughly in line with our findings.

A very different pattern was observed for maize silage. All samples were positive for the occurrence of CPFAs, with just two values falling between the LOD and the LOQ, corresponding to 93.7% of the measurements lying beyond the LOQ.

All 244 milk samples collected on farm over a 2-year period were analysed for the presence of CPFAs (Figure 2b). Similarly to what was observed for the silages, the distribution of CPFAs content in the milk of cows fed grass silage showed considerably lower values than that in the milk of cows fed maize silage. Maize silage milk had an average concentration of CPFAs of 215.8 mg kg^−1^ of fat. This was higher than the grass silage milk, which had an average of 22.1 mg kg^−1^ of fat. These results are in agreement with the previous findings on Grana Padano cheese, which showed a variable concentration of 300–830 mg kg^−1^ fat [9,32], and those of dairy products, which showed concentrations between 200 and 1000 mg kg^−1^ fat [20,32]. The LOD and LOQ reported by the authors were, respectively, 60 and 200 mg kg^−1^ of cheese fat, while in the present study, they were 8 and 26 mg kg^−1^ milk fat, respectively. For silage samples, the LOD and LOQ were 7 and 25 mg CPFAs kg^−1^ fat, whereas in the literature [15], a LOD value of 1 mg kg^−1^ DM has been reported.

Apart from two samples, one of which exhibited a CPFAs concentration below the LOD and one exhibited a CPFAs concentration between the LOD and the LOQ, hay milk was found to be negative for the marker occurrence. Maize silage milk samples were positive for CPFAs presence in 97.9% of the cases, and grass silage milk samples were positive in 79.8% of the cases (Table 3). Unexpectedly, two hay milk samples, being part of repeated measurements over time at the same farm, showed detectable CPFAs concentrations in milk (one below and one above the LOD). The results of the first experiment showed that CPFAs were still detectable 60 days after silage removal from the diet, and the CPFAs concentration of these observations decreased over time (9.65 mg kg^−1^ fat at the first sampling event, 4.37 mg kg^−1^ fat one week later and no detection in the following week). Therefore, on one hand, it cannot be excluded that these CPFAs were residual contents after a timely suspension of silage from the diet according to the hay milk guidelines, which prescribes indeed a suspension time of two weeks to formally comply with the regulations. On the other hand, Lolli et al. [20] reported that animal diet significantly affected CPFAs in ovine dairy products in particular, while the silage group showed significantly higher concentrations of CPFAs (311.2 mg kg^−1^ fat) compared to the hay group. The hay group still exhibited CPFAs with a mean value of 199.6 mg kg^−1^ fat. As already stated, CPFAs formation in microbes has been ascribed to an improvement in resistance to the acidic environment. However, DHSA forms under aerobic conditions, while LBA forms under anaerobic conditions [16]. The concentration of CPFAs has been calculated in the present work, as performed in the previous studies [9,10,11,15,20,21,28,29,30,33], as the sum of DHSA and LBA, and no differentiation between the two was done. Nevertheless, DHSA and LBA may arise from different pathways in both silage types during fermentation, but also possibly during the passage through the gastrointestinal tract of ruminants. Indeed, CPFAs were not found in pork and chicken meats [10], which are non-ruminants, whereas the phospholipid fractions isolated from sheep rumen tissues exhibited cyclopropane fatty acids [49], as did bovine heart [50].

GC-MS is regarded as a suitable approach for the screening of a large sample numbers of cheese [9]. In the present study, the method has been optimised in view of the analysis of milk. This results in an increased labour effort related to sample preparation and chromatographic analysis, making it unsuitable for large-scale screening purposes. However, it would be economically feasible for a targeted check of suspicious cases or random, infrequent checks.

### 3.3. Relationship Between Dietary CPFA Intakes from Silages and Milk CPFA Content

All correlation coefficients between the CPFAs concentration in the milk and all variables indirectly or directly describing the dietary intake of CPFAs were found to be highly significant (all <0.001) (Table 4).

The variables indirectly describing the dietary intake of CPFAs (the percentage of silage in the diet and the daily silage intake per cow, both on a dry matter basis) were found to exhibit a low correlation with the CPFAs concentration in the milk, with correlation coefficients being 0.387 and 0.431, respectively. A notable increase in the correlation coefficients with the CPFAs concentration in the milk was shown when investigating the correlation with all three variables directly quantifying the CPFAs intake (the daily CPFAs intake per cow, the daily CPFAs intake per kg of milk and the daily CPFAs intake per kg of fat produced with the milk). According to the 95% confidence intervals of the correlation coefficients, their use resulted in comparably high correlations coefficients around 0.8. This is in accordance with the expectation that not taking the very wide range of CPFAs content in the silages into account when indirectly estimating their dietary intake leads to increased uncertainty of their relationship. The multivariate exploration by principal component analysis of the relationship between the daily intake of selected feed components and several farm characteristics (milk yield, prevailing breed kept at the farm, purchase of off-farm silages, use of silage starters, occurrence of grazing at the time of milk sampling), as well as the milk fat content, the CPFAs content in the silages included in the ration, and the variables directly or indirectly quantifying the CPFAs intake with the diet, confirmed the high correlation between the three variables, directly quantifying the CPFAs intake with the diet (Figure S6). It also highlighted their close correlation with the CPFAs content in the milk. Moreover, the results of this analysis corroborate the aforementioned major role of the CPFAs concentration in the silage fat in determining the CPFAs content in the milk. No other close correlation was found with the other variables included in the analysis. An exception is represented by the dominant occurrence of the Holstein–Friesian breed in the herd, being positively correlated with the CPFAs intake with the diet and CPFAs concentration in the milk. This is presumably due to the frequent use of maize silages in the diet of this breed (in 78% of the observations, with a mean CPFAs concentration in the silage fat of 147.4 mg kg^−1^ fat ± 211.33 SD).

For the further investigation of the relationship between the dietary intake of CPFAs and CPFAs content in the milk, the daily CPFAs intake per cow and diet was used, as this is the easiest of the three variables to calculate. The other variables require additional information, like milk yield and fat content in the milk, to be computed. Given the very tight correlation of all three variables directly describing the CPFAs intake, the results obtained for the daily CPFAs intake are assumed to also be representative for the other two variables.

**Figure 3:**
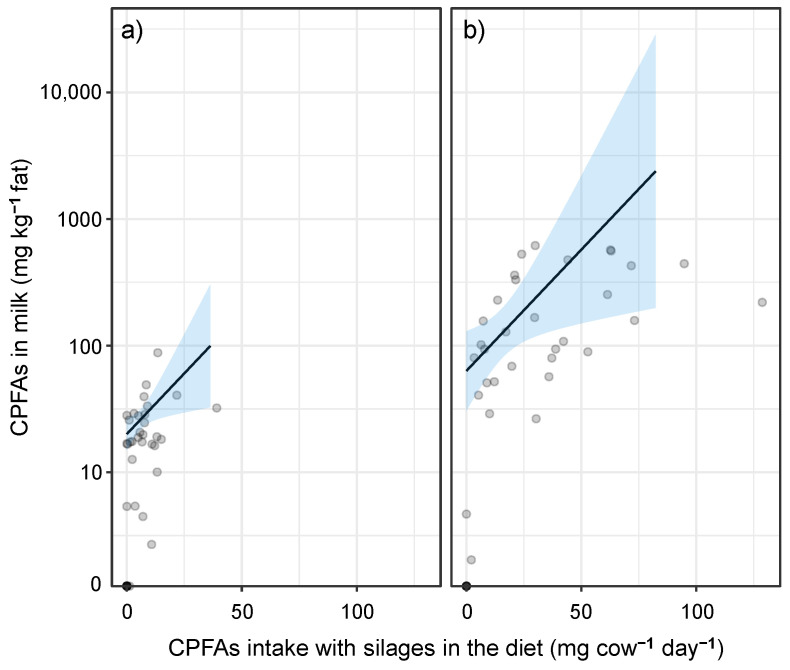
Effect of the dietary intake of CPFAs from silages (mg CPFAs cow^−1^ day^−1^) on the CPFAs content in the milk for (**a**) grass silage and (**b**) maize silage. Predicted mean values (solid black line) and the 95% confidence intervals for the true mean (shaded ribbons) based on the fixed part of the model are shown against the observed values. Overlapping observations are described by the degree of darkness on the grey scale (light grey = one observation, black = nine observations). The CPFAs content in the milk is displayed on a base-10 logarithmic scale.

The statistical analysis of the effects by means of a full model accounting for all investigated factors for the CPFAs concentration in the milk detected no seasonal effects and interactions of the investigated factors for the intake of CPFAs with the silages in the diet (Table 5). In both the full and the final model, systematically higher values were found for MSM in comparison with GSM (an estimated 3.15-fold increase for MSM) and a positive, exponential relationship was found between the CPFAs intake with the silages in the diet and the CPFAs concentration in the milk, demonstrating a quantitative relationship between dietary CPFAs input and output in the milk (a 4.5% increase in CPFA output for every extra unit of CPFA input) (Table 5, Figure 3). Unexpectedly, when maize silages are considered, the CPFAs released daily in milk per cow is, on average, much higher than the respective daily CPFAs intake (Figure S4h). It should be noted that CPFAs are reported to be negatively correlated to cis-MUFAs and odd- and branched-chain fatty acids, which are mainly associated with a low starch intake [20].

### 3.4. Sources of Uncertainty of CPFAs Prediction

At higher CPFAs intake values, there is a higher degree of uncertainty, as represented by the wider confidence intervals; this is due to the lack of data in those regions, and the fact that the variance is proportional to the mean in the inverse Gaussian model. Obtaining a predicted positive estimate for the intercept was not expected. This was due to nine observations (including repeated measurements from three of the four farms represented here) with a mean CPFAs content in the milk of 18.3 ± 9.12 SD mg kg^−1^ fat. Six of them had a relatively high silage intake (6.1 kg DM cow^−1^ day^−1^ ± 0.38 SD) and weight percentage of the whole ration (32.9% ± 3.62 SD), but no CPFAs content was detected in the silage fat, leading to an estimated CPFAs intake equal to zero.

Indeed, there are some variables used in the direct or indirect quantification of the CPFAs intake in the diet which likely produced noise and made the estimation of the relationship with the CPFAs in the milk less accurate. As the total fat content of the silage was not measured but estimated based on reference values, there was some noise in the estimated dietary intake of CPFAs from silage. Moreover, the ration was estimated using a standard tool for consultancy aims, which partly makes use of estimates from the farmer and/or of reference values instead of measurements. On the one hand, this introduces some noise in the results, but on the other hand, this reflects well the degree of precision with which the cows’ diet is described in practice. For cows grazing in summer, the quantification of the herbage intake during grazing may introduce further inaccuracy into the quantification of the effective total intake, introducing a bias (overestimation) in the percentage of silage in the diet, as well as in the amount of CPFAs ingested with it. This aspect, however, can be expected to be of minor relevance, as this case was given for just one farm within one sampling period.

## 4. Conclusions

A quantifiable CPFAs concentration above the LOQ was never observed in milk produced without the inclusion of silages in the cow’s ration; thus, its use as a marker entails no risk of false positives when no grass and maize silages are fed.

CPFAs were shown to be highly reliable in identifying maize silage usage (98%) and less reliable for grass silage (80%), with a third of the samples not quantifiable using the current analytical method. Nevertheless, the frequent simultaneous use of maize and grass silage on alpine farms might reduce the frequency of negatives if silages are used at all. On average, the concentration of CPFAs for grass silage is lower than for maize silage, and the same pattern is reported for the respective milk. To the best of our knowledge, our results demonstrate, for the first time, that there is a quantitative relationship between CPFAs intake though diet and CPFA concentration in milk.

If CPFAs are to be used as a marker in hay milk regulation, attention must be paid in the transition time from conventional milk to hay milk, especially when maize silage is included in the diet, since CPFAs were detected even after about 60 days after its removal.

The CPFA concentration in milk clearly rises, although not linearly, with an increasing dietary intake of CPFAs. However, this is less clear when only grass silages are fed, as at a low dietary intake of CPFAs, which is almost always the case when feeding grass silages alone, the increase in CPFAs in the milk is very low. This harbours the risk that, when including only grass silages in the ration, CPFAs in the milk are not detected, even with a high grass silage intake.

Future research lines deserving attention in view of the findings obtained in this study include the further investigation of time needed by CPFAs to appear in the milk after the introduction of silages in the diet; the individual determination of dihydrosterculic acid and lactobacillic acid and the investigation of their potential roles as authentication tools; the elucidation of synthesis, metabolism, and excretion of CPFAs in ruminants, possibly including experiments conducted in experimental stalls with measurements conducted on single animals, taking measurements of blood or other tissues into account.

## Figures and Tables

**Table 1:** Effect of the silage type, the time elapsed since the suspension of silage feeding in the diet of lactating dairy cows, as well as their interaction, on the concentration of CPFAs in the milk (mg kg^−1^ fat). df_n_ = numerator degrees of freedom, df_d_ = denominator degrees of freedom, F = Fisher’s F, *p* = probability, E = estimated parameter value, SE = standard error of the estimate. TSS × TSS and TSS × TSS × TSS are the second and third degree term of the polynomial, respectively. Estimates for silage type are shown for maize silage vs. grass silage.

Source	df_n_	df_d_	F	*p*	E	SE
Intercept	1	18	194.0	<0.001	24.75	4.040
Silage type (SIL)	1	4	11.1	<0.001	74.56	5.712
Time since silage suspension (TSS)	1	18	22.8	<0.008	−2.15	0.717
TSS × TSS	1	18	6.2	<0.001	0.06	0.032
TSS × TSS × TSS	1	18	2.7	<0.001	−0.01	0.001
SIL × TSS	1	18	6.7	<0.001	−5.24	1.011
SIL × TSS × TSS	1	18	1.0	0.005	0.17	0.046
SIL × TSS × TSS × TSS	1	18	1.1	0.004	−0.01	0.001

**Table 2:** Number and CPFAs content of silage samples grouped by category.

Silage Type	Number of Samples Analysed	Concentration Range of CPFAs in Positive Samples (Min–Max) ^a^	Positivity (%)
Maize silage	32	18–1292	100.0
Grass silage	35	10–461	77.1

^a^ Data reported in mg CPFAs kg^−1^ of fat extracted. LOD was set to 7 mg kg^−1^ fat. The concentration range in positive samples refers to values ≥ LOD.

**Table 3:** Number and CPFA content of milk samples grouped by category.

Milk Type	Number of Samples Analysed	Concentration Range CPFAs in Positive Samples (Min–Max) ^a^	Positivity (%)
Hay milk	48	/	4.2
Grass silage milk	99	9–186	79.8
Maize silage milk	95	23–735	97.9

^a^ Data reported in mg CPFAs kg^−1^ of fat extracted. The LOD was set to 8 mg kg^−1^ fat. The concentration range in positive samples refers to values ≥ LOD.

**Table 4:** Non-parametric Spearman’s correlation between CPFAs concentration in the milk of dairy cows (CPFA_milk_), and five variables indirectly or directly describing the dietary intake of CPFAs: Silage_%_ = percentage of silage in the diet on a dry matter basis; Silage_amount_ = mean daily silage intake per cow on a dry matter basis; CPFA_diet_ = daily CPFAs intake per cow; CPFA_diet_/kg milk = daily CPFAs intake per kg of milk; CPFA_diet_/kg fat = daily CPFAs intake per kg of fat produced with the milk. In the lower left half of the table, two-tailed *p*-values of the test are reported. In the upper right half of the table, the values of the correlation coefficient (Spearman’s ρ), as well as its 95% confidence interval (reported in brackets) are given. Spearman’s ρ values from 0 to 1 are highlighted on a white (R = 0)-to-blue (R = 1) scale.

	CPFA_milk_	Silage_%_	Silage_amount_	CPFA_diet_	CPFA_diet_/ kg Milk	CPFA_diet_/ kg Fat
CPFA_milk_	-	0.380 (0.264–0.486)	0.425 (0.313–0.526)	0.805 (0.754–0.847)	0.799 (0.747–0.842)	0.802 (0.751–0.844)
Silage_%_	<0.001	-	0.959 (0.947–0.968)	0.484 (0.378–0.577)	0.474 (0.366–0.569)	0.476 (0.369–0.571)
Silage_amount_	<0.001	<0.001	-	0.526 (0.425–0.614)	0.513 (0.410–0.602)	0.516 (0.413–0.605)
CPFA_diet_	<0.001	<0.001	<0.001	-	0.994 (0.993–0.996)	0.992 (0.990–0.994)
CPFA_diet_/ kg milk	<0.001	<0.001	<0.001	<0.001	-	0.998 (0.998–0.999)
CPFA_diet_/ kg fat	<0.001	<0.001	<0.001	<0.001	<0.001	-

**Table 5:** Parameter estimates of the fixed part of the generalised additive models for location scale and shape, analysing the effect of the investigated factors (year, season, silage type, mean CPFAs intake per cow and day) on the CPFAs content in milk for (a) the full model and (b) the final model. Analysis using means of repeated measurements over time within farm and sampling period. EST = estimate, CI = confidence interval, *p* = probability. Estimates for year are shown for the second year against the first one; those for season are shown for winter vs. summer; those for silage type are shown for milk obtained using maize silage as the only fermented feedstuff in the ration vs. milk obtained using grass silage as the only fermented feedstuff in the ration.

Term	Full Model				Final Model			
	EST	95%-CI		*p*	EST	95%-CI		*p*
		Lower Bound	Upper Bound			Lower Bound	Upper Bound	
Intercept	3.021	2.673	3.368	<0.001	2.998	2.710	3.285	<0.001
Year	−0.335	−0.747	0.078	0.116				
Season (SE)	0.090	−0.294	0.475	0.646				
Silage type (ST)	1.852	0.823	2.882	0.001	1.148	0.554	1.742	<0.001
CPFAs intake (IN)	0.044	0.006	0.081	0.025	0.044	0.008	0.081	0.021
ST × IN	−0.570	−1.505	0.366	0.237				
ST × SE	−0.020	−0.071	0.032	0.454				

## Data Availability

The original contributions presented in the study are included in the article, further inquiries can be directed to the corresponding author.

## Supplementary Materials

The following supporting information can be downloaded at: https://www.mdpi.com/article/10.3390/foods14030476/s1, Figure S1: Percentage of maize and grass silage in the dry matter of the total ration at the farms sampled in the summer and winter season in the two observation years; Figure S2: Map of South Tyrol (Northern Italy) showing the location of the farms sampled during the project; Table S1: Characteristics of the farms sampled for milk and silages; Figure S3: Change in CPFAs concentration in milk of dairy cows after the introduction of maize silage as the only fermented feedstuff in the cow’s ration of a South Tyrolean farm; Figure S4: Distribution of the variables used to directly or indirectly quantify the mean dietary daily intake of CPFAs from silages; Figure S5: Scatterplot of all valid observations of dietary CPFAs intake from (a) grass silages and (b) maize silages and CPFAs concentration in the milk; Figure S6: Biplot of the first two principal components from a PCA describing the farm characteristics, the intake of crude ashes, crude protein, fibre, P, and Fe through the feed ration, the variables used to indirectly or directly used to quantify the dietary CPFAs intake from silages and the CPFAs in the milk of farms feeding silages.

## Abbreviations

The following abbreviations are used in this manuscript:

CPFAs	Cyclopropane fatty acids
DHSA	Dihydrosterculic acid
FA	Fatty acid
FAME	Fatty acid methyl esters
GS	Grass silage
GSM	Grass silage milk
GC-MS	Gas chromatography–mass spectrometry
HM	Hay milk
LAB	Lactic acid bacteria
LBA	Lactobacillic acid
MS	Maize silage
MSM	Maize silage milk
