# Supplementary material for: Effect of Diet on CPFAs Used as Markers in Milk for the Detection of Silage in the Ration of Dairy Cows
Source: Foods. 2025 Feb 2;14(3):476. doi: 10.3390/foods14030476 (PMC11816875; doi:10.3390/foods14030476)
Supplement: Supplementary file 1 [file foods-14-00476-s001.zip › foods-3415259-supplementary.pdf]

foods

MDPI

Supplementary Materials

Effect of the diet on CPFAs used as markers in milk for the detection of silage in the ration of dairy cows

Federico Fava[1], Demian Martini-Lösch[1], Giovanni Peratoner[1*], Peter Robatscher[1], Aldo Matteazzi[1], Evelyn Soini[1], Andreas Österreicher[2], Simon Volgger[3,§], Rafael de Andrade Moral[4], Matteo Mario Scampicchio[5] , Daniela Eisenstecken[1], Elena Venir[1]

[1] Laimburg Research Centre, Laimburg 6 – Pfatten (Vadena), 39040 Auer (Ora), BZ, Italy
[2] Sennereiverband Südtirol, Via Galvani 38, 39100 Bozen-Bolzano, Italy
[3] Beratungsring Berglandwirtschaft, Via Galvani 38, 39100 Bozen-Bolzano, Italy,
[4] Department of Mathematics & Statistics, Maynooth University, Maynooth, Ireland
[5] Faculty of Agricultural, Environmental and Food Sciences, Free University of Bozen-Bolzano, Piazza Università 5, 39100 Bozen-Bolzano, Italy
[§] Present address: Untere Gasse 10, 39040 Ratschings, BZ, Italy
*Correspondence: giovanni.peratoner@laimburg.it; Tel. +39-0471-969661

Table of contents

Figure S1: Percentage of maize and grass silage in the dry matter of the total ration at the farms sampled in the summer and winter season in the two observation years……………………………………………………………………………….2

Figure S2: Map of South Tyrol (Northern Italy) showing the location of the farms sampled during the project…………3

Table S1: Characteristics of the farms sampled for milk and silages……………………………………………………….4

Figure S3: Change of CPFAs concentration in milk of dairy cows after the introduction of maize silage as the only fermented feedstuff in the cow's ration of a South Tyrolean farm……………………………………………………………5

Figure S4: Distribution of the variables used to directly or indirectly quantify the mean dietary daily intake of CPFAs from silages……..6

Figure S5: Scatterplot of all valid observations of dietary CPFAs intake from a) grass silages and b) maize silages and CPFAs concentration in the milk………………………………………………………………………………………………..7

Figure S6: Biplot of the first two principal components from a PCA describing the farm characteristics, the intake of crude ashes, crude protein, fibre, P and Fe through the feed ration, the variables used to indirectly or directly used to quantify the dietary CPFAs intake from silages and the CPFAs in the milk of farms feeding silages………………………8

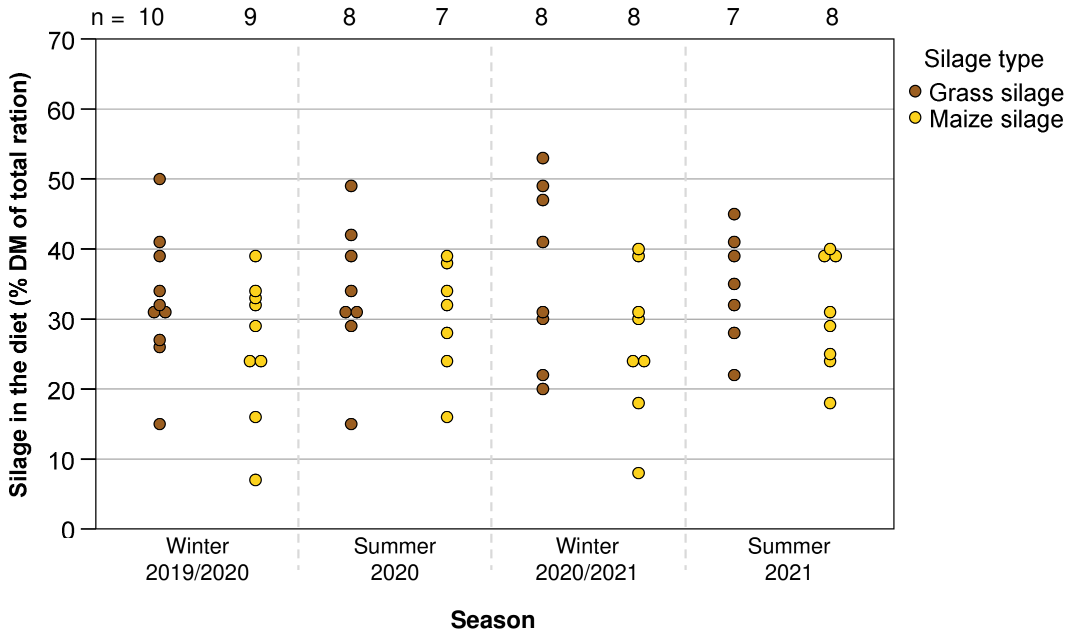

Figure S1. Percentage of maize and grass silage in the dry matter of the total ration at the farms sampled in the summer and winter season in the two observation years.

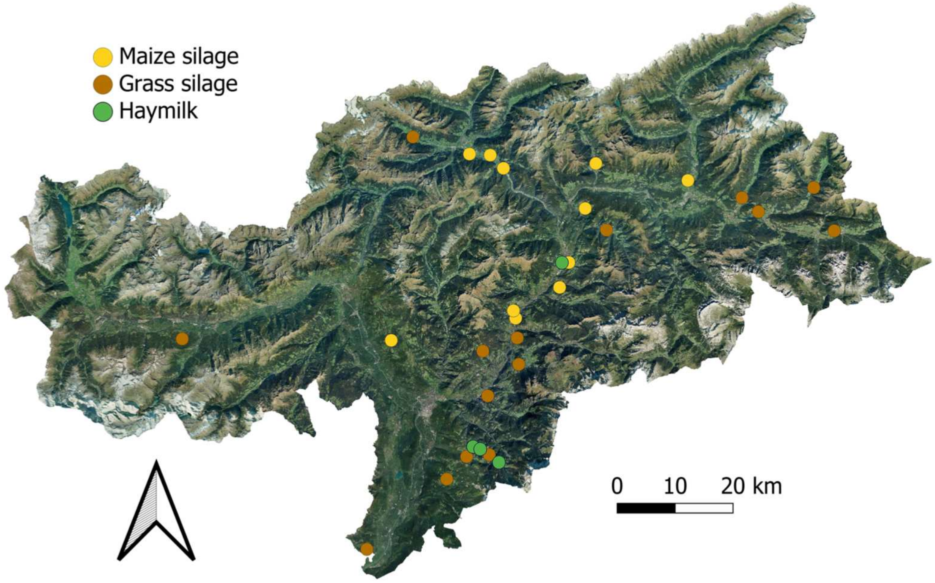

Figure S2. Map of South Tyrol (Northern Italy) showing the location of the farms sampled during the project. Farms in which haymilk (HM) was sampled are shown in green, those in which grass silage milk (GSM) was sampled are shown in brown and those in which maize silage milk (MSM) was sampled are shown in yellow.

Table S1. Characteristics of the farms sampled for milk and silages.

		Milk type					
		Haymilk		Grass silage milk		Maize silage milk	
Feeding period		Summer	Winter	Summer	Winter	Summer	Winter
Observations (n)		8	8	15	18	15	17
Farmstead elevation (m a.s.l.)		1173±353.3 [602-1402]	1173±353.3 [602-1402]	1204±201.9 [863-1470]	1208±219.3 [748-1470]	874±151.8 [542-1036]	927±144.3 [616-1131]
Breed	Brown (%)	24.0±44.51 [0.0-96.2]	24.0±44.51 [0.0-96.2]	29.7±36.66 [0.0-100.0]	37.9±40.63 [0.0-100.0]	10.2±25.86 [0.0-100.0]	14.8±32.73 [0.0-100.0]
	Jersey (%)	0.0±0.00 [0.0-0.0]	0.0±0.00 [0.0-0.0]	1.3±3.35 [0.0-9.5]	0.5±2.11 [0.0-8.7]	0.0±0.00 [0.0-0.0]	0.0±0.00 [0.0-0.0]
	Simmental (%)	2.3±4.21 [0.0-9.1]	2.3±4.21 [0.0-9.1]	28.2±44.99 [0.0-100.0]	20.2±34.39 [0.0-100.0]	18.4±34.32 [0.0-87.0]	22.5±38.66 [0.0-92.5]
	Holstein-Friesian (%)	46.4±48.08 [0.0-100.0]	46.4±48.08 [0.0-100.0]	39±37.27 [0.0-100.0]	34.1±36.39 [0.0-100.0]	69±39.96 [0.0-100.0]	49.9±44.19 [0.0-100.0]
	Tyrolean Grey (%)	27.3±45.06 [0.0-100.0]	27.3±45.06 [0.0-100.0]	1.9±5.03 [0.0-14.3]	7.3±15.89 [0.0-47.6]	0.0±0.00 [0.0-0.0]	11.8±33.21 [0.0-100.0]
	Cross-breeds and other breeds (%)	0.0±0.00 [0.0-0.0]	0.0±0.00 [0.0-0.0]	0.0±0.00 [0.0-0.0]	0.0±0.00 [0.0-0.0]	2.4±6.40 [0.0-18.2]	1.1±4.41 [0.0-18.2]
Breeds kept at the farm (n)		1.75±0.886 [1-3]	1.75±0.886 [1-3]	1.73±0.884 [1-3]	1.88±0.697 [1-3]	1.67±0.724 [1-3]	1.65±0.702 [1-3]
Farms keeping more than one breed (%)		50.0	50.0	46.7	66.7	53.3	52.9
Herd size (n of milking cows)		26.5±4.31 [22-33]	26.5±4.31 [22-33]	25.7±9.42 [13-40]	26.5±9.64 [13-40]	40.3±32.34 [14-110]	34.2±25.07 [15-110]
Milk yield x 1000 (kg cow^{-1} year^{-1})		7.50±1.134 [6.0-8.5]	7.25±9.636 [6.0-8.5]	8.03±1.111 [6.5-10.00]	7.70±1.186 [6.0-9.5]	8.59±8.484 [7.5-10.0]	8.35±1.344 [5.5-10.0]
Farms with grazing (%)		50.0	0.0	20.0	0.0	20.0	0.0
Grazing period	pre-grazing (%)	25.0	0.0	0.0	0.0	0.0	0.0
	spring grazing (%)	50.0	0.0	0.0	0.0	0.0	0.0
	summer grazing (%)	50.0	0.0	0.0	0.0	6.7	0.0
	autumn grazing (%)	25.0	0.0	80.0	0.0	13.3	0.0

All values are means ± standard deviation except those of 'Farms with grazing', 'Farms keeping more than one breed' and 'Grazing period' (percent of observations). Ranges are given in square brackets.

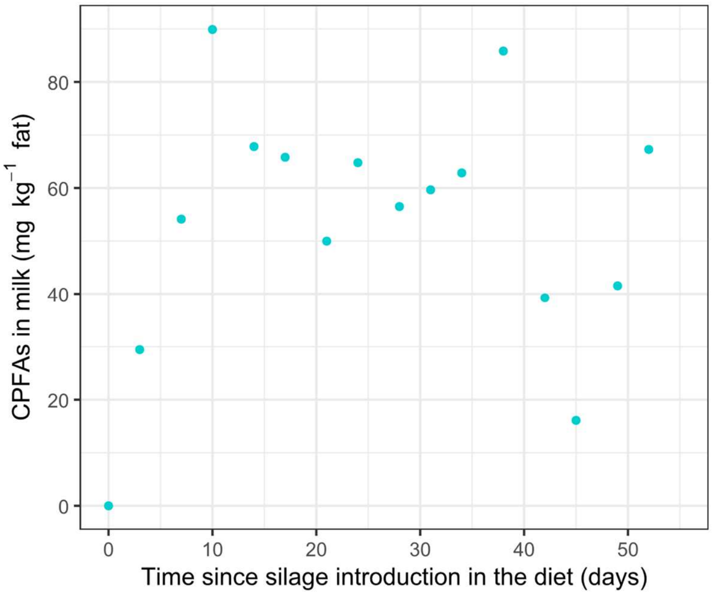

Figure S3. Change of CPFAs concentration in milk of dairy cows after the introduction of maize silage as the only fermented feedstuff in the cow's ration of a South Tyrolean farm. Milk samples were taken twice a week (every 3 to 4 days) for 2 months at a farm switching from haymilk feeding to a conventional regime, for a total of 32 samples. The first sampling occurred on the first day in which the new ration was fed. See the main text for details about the sampling procedure.

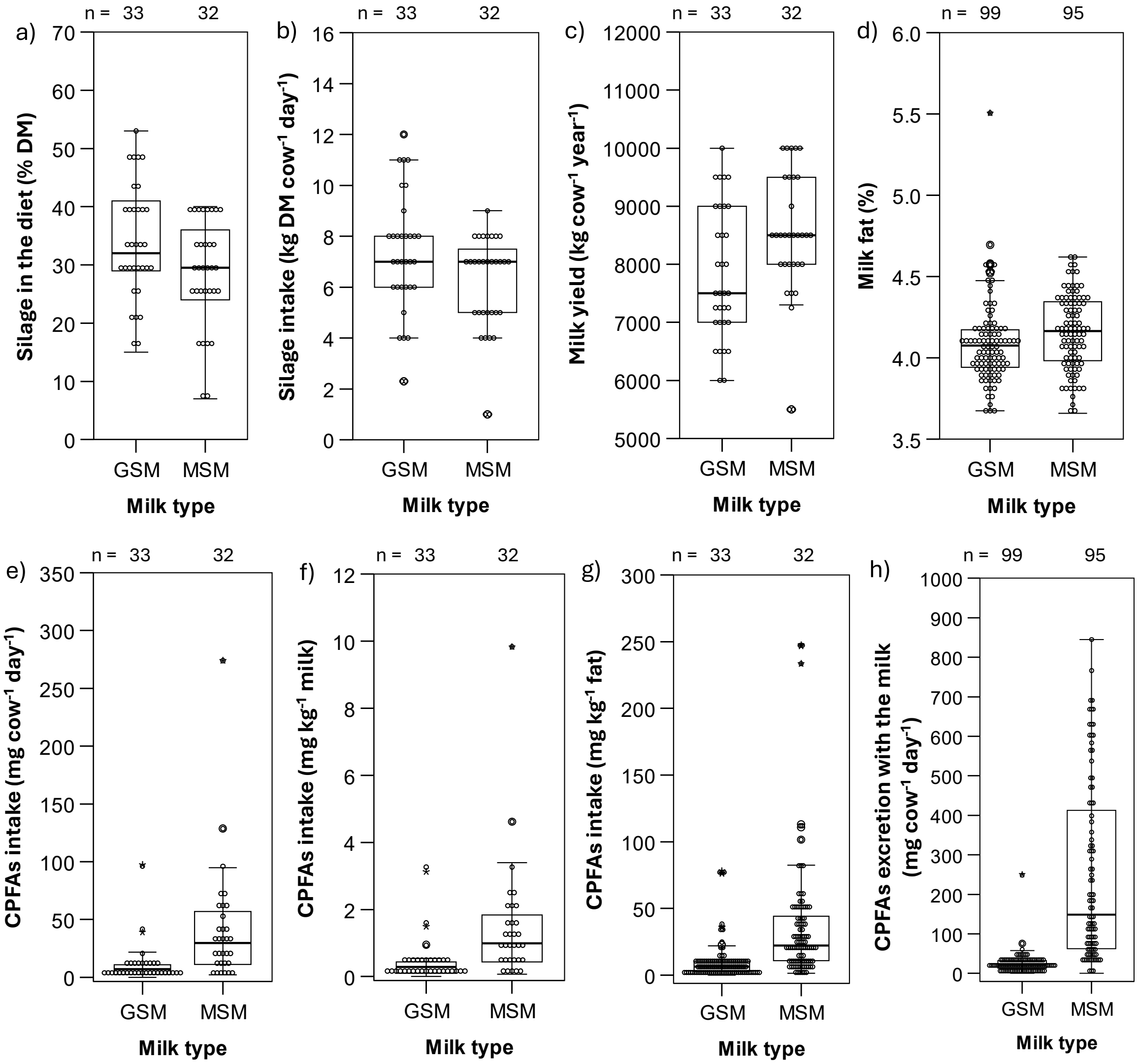

Figure S4. Distribution of the variables used to directly or indirectly quantify the mean dietary daily intake of CPFAs from silages: a) percentage of silage in the diet on dry matter basis, b) mean daily silage intake per cow on dry matter basis, c) milk yield, d) milk fat content, e) daily dietary intake of CPFAs from silages per cow, f) dietary intake of CPFAs from silages per kg of milk and g) dietary intake of CPFAs from silages per kg of fat produced with the milk. The CPFAs content in the silages is reported in Figure 2.The data is represented by means of boxplots. n = number of observations, GSM = milk produced including grass silages as the only fermented feedstuff in the ration, MSM = milk produced including maize silages as the only fermented feedstuff in the ration.

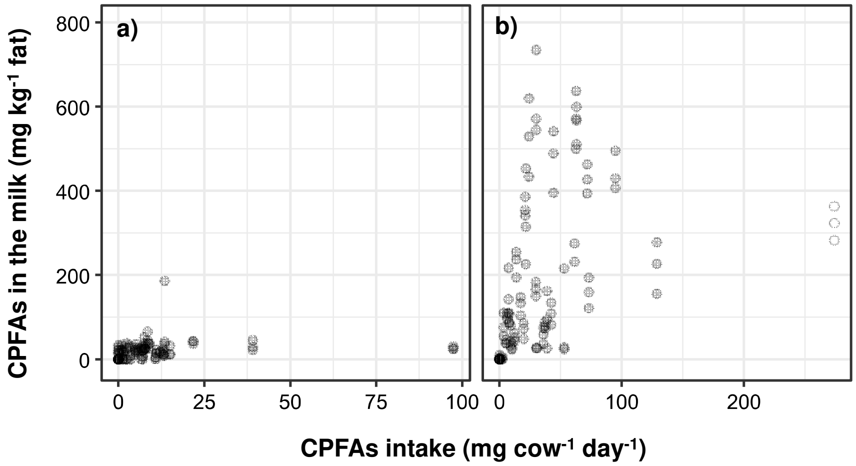

Figure S5. Scatterplot of all valid observations of dietary CPFAs intake from a) grass silages and b) maize silages in the diet and CPFAs content in the milk, including repeated measurements over time at the same farm within the same sampling period (combination of year and season). Overlapping observations are described by the degree of darkness on the grey scale (light grey = 1 observation, black = 29 observations). Three observation not having been used for the formal analysis of the effect of CPFAs intake on CPFAs in the milk (see Table 5 and Figure 4 in the main paper) are shown in white colour.

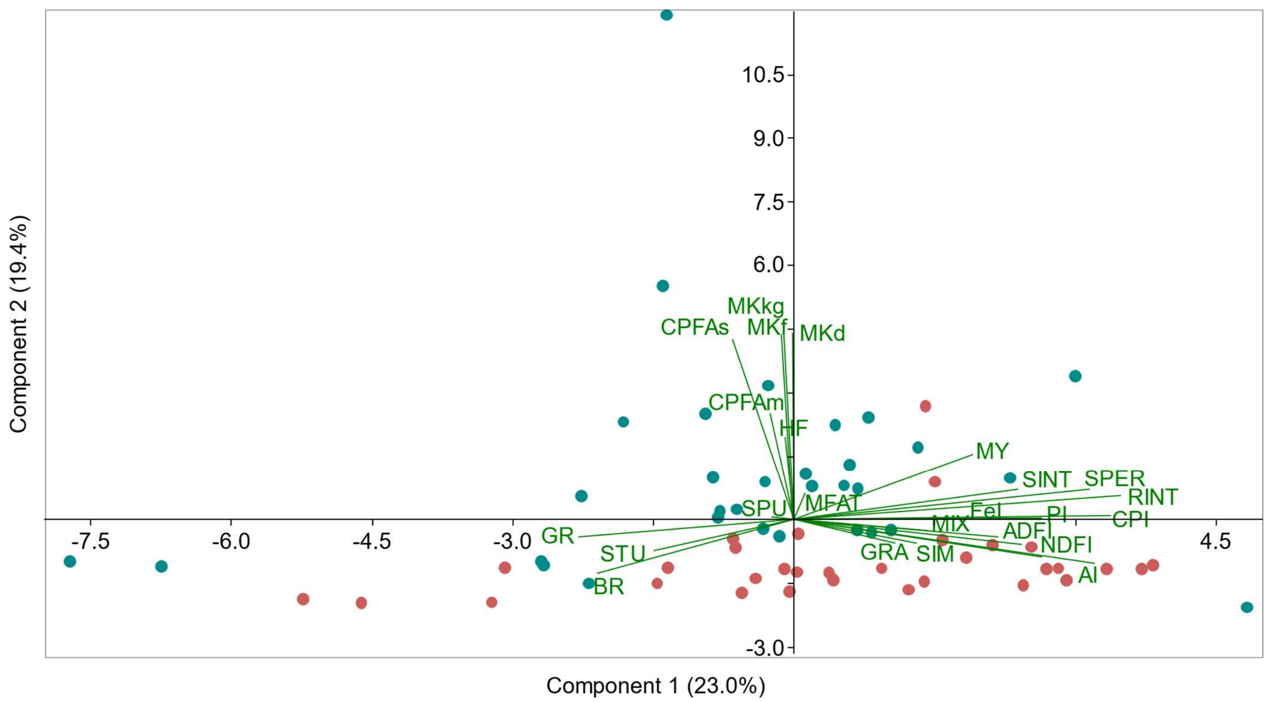

Figure S6. Biplot of the first two principal components from a PCA describing the farm characteristics, the intake of crude ashes, crude protein, fibre, crude protein, P, Fe through the feed ration, the variables used to indirectly or directly used to quantify the dietary CPFAs intake from silages and the CPFAs in the milk of farms feeding silages. AI = daily crude ashes intake per cow, CPI = daily crude protein intake per cow, NDFI = daily NDF intake per cow, ADFI = daily ADF intake per cow, PI = daily phosphorous intake per cow, FeI = daily iron intake per cow, MY = annual milk yield per cow, BR = Brown as prevailing breed in the herd (>90% of the total cows number, binary scaled), SIM = Simmental as prevailing breed in the herd (>90% of the total cows number, binary scaled), HF = Holstein-Friesian as prevailing breed in the herd (>90% of the total cows number, binary scaled), GR = Tyrolean Grey as prevailing breed in the herd (>90% of the total cows number, binary scaled), MIX = no prevailing breed in the herd (binary scaled), SPU =purchase of off-farm silage (binary scaled), STU = use of silage starter (binary scaled), GRA = occurrence of grazing at the time of milk sampling (binary scaled), MFAT = milk fat (%), CPFAs = cyclopropane fatty acids in the silage (mg kg^{-1} fat), RINT = daily total ration intake per cow, SINT =daily silage intake per cow, SPER = percentage of silage in the diet on dry matter basis (%), MKd = CPFAs intake with the silage in the diet per cow, MKkg = CPFAs intake with the silage in the diet per kg of milk, MKf = CPFAs intake per kg of fat produced with the milk, CPFAm = cyclopropane fatty acids in the milk (mg kg^{-1} fat). The vectors of CPFAm, MKkg and HF have been slightly translated to allow them to be distinguished by those od CPFAs, MKf and MKkg respectively. Blue points (n=32) are of observation at farms feeding maize silage as only fermented feedstuff in the feed ration, red points (n=33) are observations at farms feeding grass silage as only fermented feedstuff in the feed ration.
